# COG5-CDG: expanding the clinical spectrum

**DOI:** 10.1186/1750-1172-7-94

**Published:** 2012-12-10

**Authors:** Daisy Rymen, Liesbeth Keldermans, Valérie Race, Luc Régal, Nicolas Deconinck, Carlo Dionisi-Vici, Cheuk-wing Fung, Luisa Sturiale, Claire Rosnoblet, François Foulquier, Gert Matthijs, Jaak Jaeken

**Affiliations:** 1 Centre for Human Genetics, University of Leuven, Leuven, Belgium; 2 Centre for Metabolic Diseases, University Hospital Gasthuisberg, Herestraat 49, , BE, -3000, Leuven, Belgium; 3 University Children’s Hospital Queen Fabiola, Brussels, Belgium; 4 Division of Metabolism, Bambino Gesù Hospital, Rome, Italy; 5 Duchess of Kent Children’s Hospital, University of Hong Kong, Pokfulam, Hong Kong; 6 Institute of Chemistry and Technology of Polymers, Catania, Sicily; 7 Structural and Functional Glycobiology Unit, University of Lille 1, Lille 1, France

**Keywords:** CDG-II, Glycosylation, Glycan analysis, Conserved oligomeric golgi complex, COG5, Trafficking

## Abstract

**Background:**

The Conserved Oligomeric Golgi (COG) complex is involved in the retrograde trafficking of Golgi components, thereby affecting the localization of Golgi glycosyltransferases. Deficiency of a COG-subunit leads to defective protein glycosylation, and thus Congenital Disorders of Glycosylation (CDG). Mutations in subunits 1, 4, 5, 6, 7 and 8 have been associated with CDG-II. The first patient with COG5-CDG was recently described (Paesold-Burda *et al*. Hum Mol Genet 2009; 18:4350–6). Contrary to most other COG-CDG cases, the patient presented a mild/moderate phenotype, i.e. moderate psychomotor retardation with language delay, truncal ataxia and slight hypotonia.

**Methods:**

CDG-IIx patients from our database were screened for mutations in COG5. Clinical data were compared. Brefeldin A treatment of fibroblasts and immunoblotting experiments were performed to support the diagnosis.

**Results and conclusion:**

We identified five new patients with proven COG5 deficiency. We conclude that the clinical picture is not always as mild as previously described. It rather comprises a broad spectrum with phenotypes ranging from mild to very severe. Interestingly, on a clinical basis some of the patients present a significant overlap with COG7-CDG, a finding which can probably be explained by subunit interactions at the protein level.

## Background

Proteins can undergo different forms of post-translational modification within the endoplasmic reticulum (ER) and the Golgi. Around 70% of all proteins are glycosylated. Glycoproteins serve many critical roles in metabolism, including protein folding, cell recognition, cell adhesion… The importance of the glycosylation pathway is illustrated by a group of diseases termed Congenital Disorders of Glycosylation (CDG). Patients present a broad clinical spectrum. Usually there is multi-system involvement, though neurological symptoms and dysmorphic features often dominate the clinical picture. To date, many types of CDG involving defects in the biosynthesis, transfer and remodelling of the N-glycan have been discovered. Since at least 200–300 genes are known to be involved in glycosylation (~1% of the human genome), one expects that the knowledge of CDG and diagnosis today represents only the tip of the iceberg. Most types of CDG are caused by mutations in genes directly involved in the glycosylation pathway, i.e. glycosyltransferases, glycosidases or nucleotide sugar transporters. However, a growing number of protein deficiencies which indirectly affect glycosylation are reported, for example defects in the Conserved Oligomeric Golgi (COG) complex [1].

The COG complex consists of eight subunits, organized in lobe A (COG1 to COG4) and lobe B (COG5 to COG8). The two lobes are bridged by an interaction between COG1 and COG8. The complex is involved in retrograde vesicle transport within the Golgi, thereby affecting the localization of GEAR proteins (i.e. glycosyltransferases, glycosidases, golgin and SNARE proteins). Correct glycosylation requires that the different proteins involved are distributed in a defined gradient across the Golgi cisternae. In patients with COG deficiency the localization of GEAR proteins is disturbed, leading to default glycosylation and thus CDG. Interestingly, it seems that GEAR protein levels are influenced in a different way by defects in lobe A versus lobe B. Until now it appears that GEARs residing in the early Golgi cisternae (e.g. mannosidase II) are more affected when a lobe A subunit is deficient, while GEARs residing in the late Golgi cisternae (e.g. galactosyltransferases and sialyltransferases) are more influenced by lobe B alterations. Furthermore, lobe A alterations cause important changes in Golgi structure leading to accumulation of late glycosylation enzymes in CCD (COG complex-dependent) vesicles, thereby preventing interaction with their substrate, whereas lobe B deficiency mainly results in altered steady state levels of these enzymes due to their translocation to the ER and subsequent proteasomal degradation [2-7].

Since retrograde trafficking is disturbed in COG deficient cells, a delay in Golgi disruption will be seen upon treatment of cells with Brefeldin A (BFA). BFA is an inhibitor of the GDP/GTP-exchange factor for ADP-ribosylation factor 1. So, upon treatment coat proteins will be released from Golgi membranes. This leads to formation of tubule-like structures and a rapid collapse of the Golgi into the ER due to retrograde transport. Using a Golgi protein (e.g. ß1,4 galactosyltransferase 1), one can visualize its redistribution from the Golgi to the ER during BFA treatment. A delay in this process will be seen in COG deficient cells [2].

Mutations in the genes of six different COG-subunits have been reported, i.e. COG1 and COG4 to COG8 [8-18]. The first patient with COG5-CDG was only recently published [12]. The patient presented a mild/moderate phenotype, with moderate psychomotor retardation, language delay, truncal ataxia and slight hypotonia. Here we present five additional patients with proven COG5 deficiency.

## Methods

### Glycoprotein analysis

IEF of serum transferrin was performed using the method described by Carchon *et al.*[19]. Further delineation of the glycan structure was obtained by matrix assisted laser desorption/ionization mass spectrometry (MALDI-TOF MS) of serum transferrin. After PNGase treatment, the released N-glycans were purified by solid-phase extraction and permethylated in the presence of sodium hydroxide. The permethylated glycans were analyzed by MALDI-TOF MS in negative and positive ion mode [20,21].

### Molecular analysis

Mutation analysis was performed on genomic DNA for all patients. Mutation analysis on cDNA was performed for patients 3 and 4. Total RNA was extracted from fibroblasts by using the RNeasy Mini Kit (Qiagen, Venlo, The Netherlands) according to the manufacturer’s protocol. To remove residual traces of genomic DNA, the RNA was treated with DNase I (Qiagen, Venlo, The Netherlands) while bound to the RNA binding column. The concentration and purity of the RNA was measured using a Nanodrop ND-1000 spectrophotometer (Thermo Scientific, Aalst, Belgium). RNA was reverse transcribed into cDNA by using the First-strand cDNA Synthesis Kit (GE Healthcare, Diegem, Belgium) according to the manufacturer’s protocol. In patient 2, 3 and 4 analysis was performed through Sanger sequencing (primers available on demand). Amplification conditions were 2 min at 95°C, 10 cycles of 30s at 95°C, 30s at 65°C (−1°C each cycle), 2 min at 72°C followed by 25 cycles of 30s at 95°C, 30s at 55°C, 4 min at 72°C. Purification of the PCR product was performed by adding ExoSAP-IT (Isogen, De Meern, The Netherlands), followed by incubation of the product for 15 min at 37°C and 15 min at 80°C. Sequence analysis was performed using universal M13 primers (Integrated DNA Technologies, Leuven, Belgium), Big Dye Terminator V3.1 Kit Cycle Sequencing and 5X Sequencing Buffer (Applied Biosystems, Gent, Belgium). Sequencing conditions were 3 min at 96°C followed by 25 cycles of 10s at 96°C, 5s at 50°C, 4 min at 60°C. Precipitation of the product was performed with the aid of the Biomek NX^p^ (Beckman Coulter, Suarlée, Belgium). Mutation analysis was carried out on an ABI3130xl automated sequence detection system (Applied Biosystems, Gent, Belgium).

In patients 1.1 and 1.2, the identification of the mutations was performed by whole exome sequencing, using the Nimblegen Exome Capturing Kit version 2 (Roche, Vilvoorde, Belgium). Sequencing was performed on the HiSeq2000 (Illumina, Eindhoven, The Netherlands).

### Western blot analysis of the COG5 and COG7 subunits

Control and patients fibroblasts were rinsed twice with ice cold PBS and lysed on ice for 30 min in lysis buffer (Ripa: 25 mM Tris pH 7.6, 150 mM NaCl, 1% NP40, 1% sodium deoxycholate, 0.1% sodium dodecyl sulfate) with protease inhibitor cocktail (Roche, Vilvoorde, Belgium). Insoluble material was removed by centrifugation for 30 min at 20,000 g at 4°C. Proteins were quantified using Micro BCA protein assay kit (Thermo Scientific, Aalst, Belgium). Equal quantities (15 μg) of protein were mixed with a reducing LDS loading buffer containing DTT (Invitrogen). The mixture was incubated 3 min at 100°C. Proteins were separated on 4–12% Bis-Tris gels (Invitrogen), and transferred onto a nitrocellulose membrane (GE Healthcare, Diegem, Belgium). Non-specific binding sites were blocked by incubating the membrane in TBS containing 0.05% Tween-20 (TBS-T) and 5% non-fat dried milk for 1 hour at room temperature. The membrane was then incubated for 1 hour with the primary rabbit antibody dissolved in blocking buffer. Anti-COG5 and COG7 antibodies were gifts from D. Ungar (Princeton University, Princeton, USA) and M. Krieger (Massachusetts Institute of Technology, Cambridge, USA) respectively. After washing in TBS-T, horseradish peroxidase-linked secondary goat anti-rabbit antibody (P0448, Dako; used at a dilution of 1:10.000) was applied to the membrane. Signals were detected using chemiluminescence reagent (ECL, PerkinElmer, Zaventem, Belgium) on imaging film (GE Healthcare, Diegem, Belgium). Signal detection was performed by autoradiography and quantified with the ImageQuant LAS 4000 software (GE Healthcare, Piscataway, NJ, USA).

### BFA treatment of cells

Fibroblasts were grown overnight on glass bottom culture dishes (MatTek Corporation, Ashland, USA) and transfected with pcDNA3.1 encoding GalT-GFP. Transfected cells were visualized by confocal microscopy. The BFA assay was subsequently performed. The medium replaced by medium containing 5 μg/ml BFA. Time-lapse images were taken with a Leica Sp5 microscope. A total of 50 images were acquired at the rate of 1/10s (0.3s exposure). The fluorescence was monitored by imaging the Golgi region.

## Results

### Patient description

The clinical features of the 6 patients and of the index patient are summarized in Table 1.

**Table 1:** Clinical features of COG5-CDG patients

	**Index**	**P1.1**	**P1.2**	**P1.3**	**P2**	**P3**	**P4**
**Ethnic origin**	Iraqi	Moroccan	Moroccan	Moroccan	Chinese	Italian	Belgian
**Consanguinity**	+	+	+	+	-	+	-
**Mental retardation**	Moderate	Moderate	Severe	Severe	Mild	Severe	Severe
**Delayed speech development**	+	++	++	++	+	++	++
**Delayed motor development**	+	++	++	++	+	++	++
**Cerebral/cerebellar atrophy**	Cerebellar	-	NA	-	-	Both	-
**Microcephaly**	+	+	+	+	-	++	++
**Hypotonia**	+	+	+	+	+	++	++
**Convulsions**	-	-	-	-	-	-	+
**Short stature**	-	+	+	+	-	+	+
**Liver involvement**	-	-	-	-	+	+	-
**Deafness**	-	-	-	-	-	+	+
**Blindness**	-	-	-	-	-	+	+
**Neurogenic bladder**	-	-	-	-	-	+	+
**Other**		-	-	-	-	Contractures	-	Wrinkled skin

NA: not available.

Patient 1.1, patient 1.2 and patient 1.3 are siblings. They were born in a family of seven children from consanguineous Moroccan parents. Two nephews are known to have mental retardation of unknown origin.

**Figure 1:**
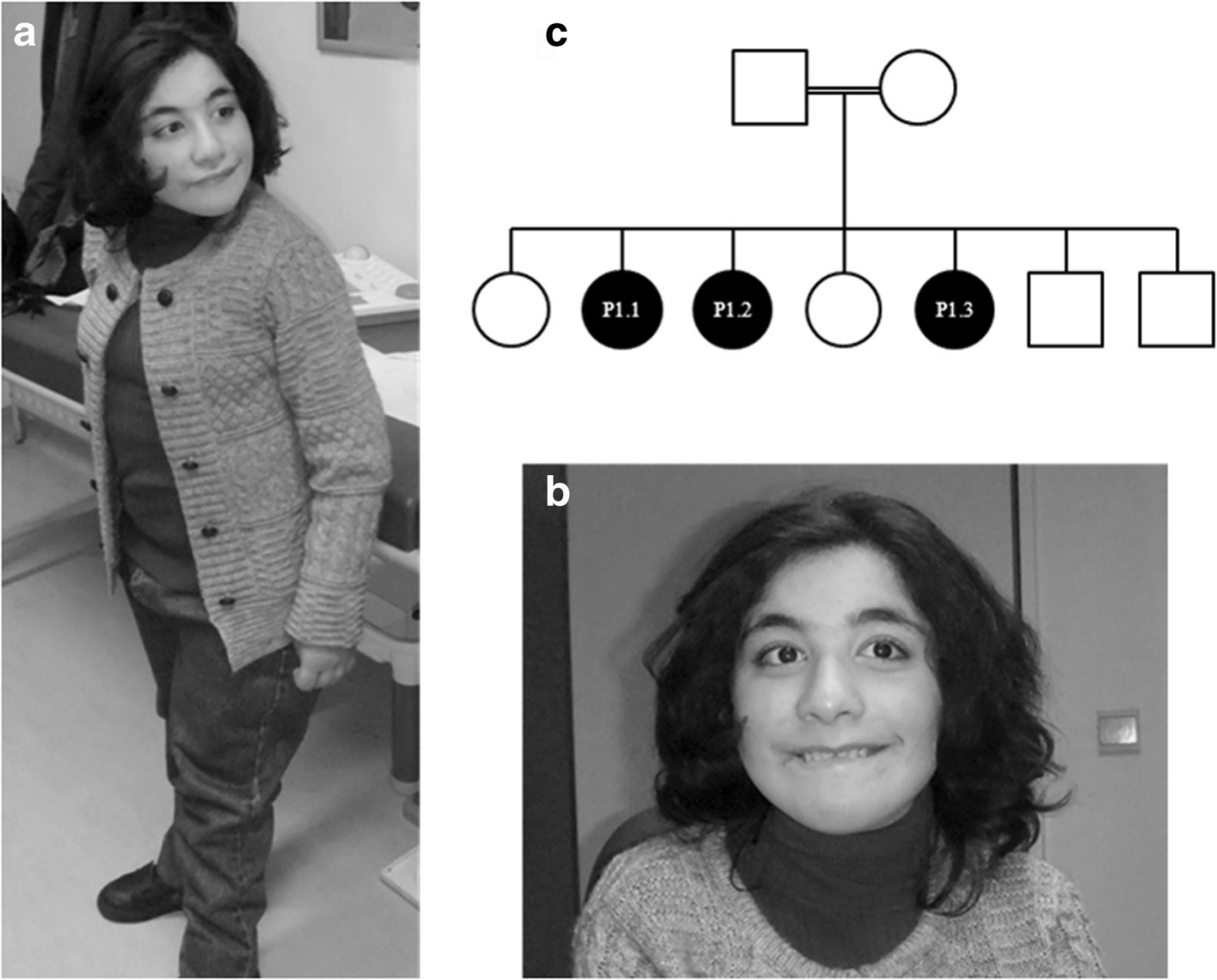
**Clinical features of patient 1.1 at the age of 15.** Note the short stature (**a**), strabismus, prominent nose, thin upper lip and microcephaly (**b**). Family tree of patients 1.1, 1.2 and 1.3 (**c**).

Patient 1.1 is a 15 year old girl (Figure 1). The pregnancy was unremarkable and she was born at term. General developmental delay and hypotonia was noted after the age of 1 year, with subsequent delays in fine motor and language development. At the age of 8 years she spoke her first words, but she never constructed sentences. Now, at the age of 15, she communicates with simplified sign language. Ophthalmologic examination and hearing tests were normal. Psychological evaluation could not retain signs of autism. Brain MRI at the age of 10 years revealed a global decrease of white matter and enlarged lateral ventricles. To date, she presents with short stature, moderate mental retardation and non-progressive microcephaly. Slight dysmorphic features are present, i.e. posteriorly rotated, low set ears, a prominent nose and low hair line. Menarche occurred at the age of 13. She has genua valga and a wide based gait. Running is easier for her than walking. Reflexes are brisk.

Patient 1.2 is a 19 year old girl. Birth was complicated by umbilical cord strangulation. Her global development was severely delayed, and language development did not occur. At the age of 6 years she learned to walk independently. Ophthalmologic examination and hearing tests were normal. To date she presents with severe mental retardation, slight dysmorphism and autistic behavior. She can only walk short distances; otherwise the use of a wheelchair is required. She is unable to dress herself or to eat independently. There is urinary incontinence.

Patient 1.3 is a 28 year old woman. She was born at term. Pregnancy was complicated with premature contractions at 12 weeks gestational age. Developmental delay was noted at the age of 8 months. She presented with hypotonia and a delay in motor development. At the age of 3 she learned to pronounce simple words, but there was no further language development. She was able to walk independently at the age of 5 years. Hearing tests were normal. Ophthalmologic examination showed strabismus due to hyperopia. Brain imaging did not show any signs of cerebral atrophy. Actually, she presents with non-progressive microcephaly, severe mental retardation (IQ < 20) and autistic behavior. She also has slight dysmorphic features, i.e. low set, posteriorly rotated ears and a high arched palate. She is dependent of her environment for daily activities. She is only able to walk with support. There is urinary incontinence.

Patient 2 is a girl of 9 years old (previously described by Fung *et al*. JIMD Reports 2012;3:67–70). She is the second child of healthy, non-consanguineous parents of Chinese origin. Pregnancy was complicated by intra-uterine growth retardation (IUGR). A caesarean section was performed at the gestational age of 35 weeks due to oligohydramnios. At the age of 8 months she presented with hypotonia, failure to thrive, microcephaly, general developmental delay, hepatosplenomegaly and flexion contractures of all fingers. There was no facial dysmorphism. Further investigations revealed cirrhosis with portal hypertension. No infectious, autoimmune or metabolic cause of the liver disease was found. Brain MRI at the age of 13 months showed delayed myelination. Hearing and ophthalmological tests were normal. A progressive improvement of developmental milestones occurred. Now she presents with non-progressive microcephaly and mild mental retardation (IQ 62).

**Figure 2:**
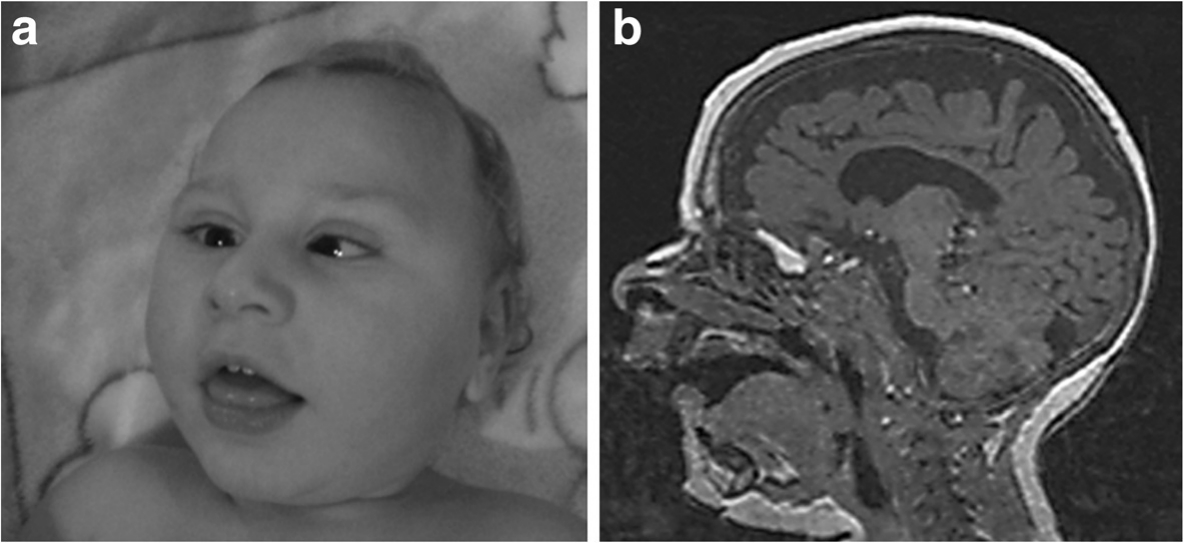
**Clinical features and brain MRI of patient 3 at the age of 12 months.** Note the important microcephaly, facial hypotonia, retrognathia and strabismus (**a**). MRI shows global cerebral and cerebellar atrophy (**b**).

Patient 3 is a 3 year old boy (Figure 2). He is the first child of consanguineous, Italian parents. During pregnancy IUGR was noted. He was born at term by caesarean section because of breech presentation. Body weight and head circumference were at the third percentile, but length was found far below the third percentile. At 3 months of age, the child was hospitalized because of important hypotonia, progressive microcephaly, failure to thrive, strabismus, recurrent urinary tract infections and hepatomegaly. TORCH screening was negative. Investigations revealed important liver involvement and a neurogenic bladder. Brain MRI at the age of 1 year showed severe supra- and subtentorial brain atrophy (Figure 2). Hearing tests and ophthalmological examination demonstrated the presence of sensorineural deafness and cortical blindness respectively. To date the child presents with severe mental retardation, spastic quadriplegia and scoliosis. There is no language development. Because of poor feeding a gastrostomy was performed. Neurogenic bladder dysfunction was treated with a cystostomy.

**Figure 3:**
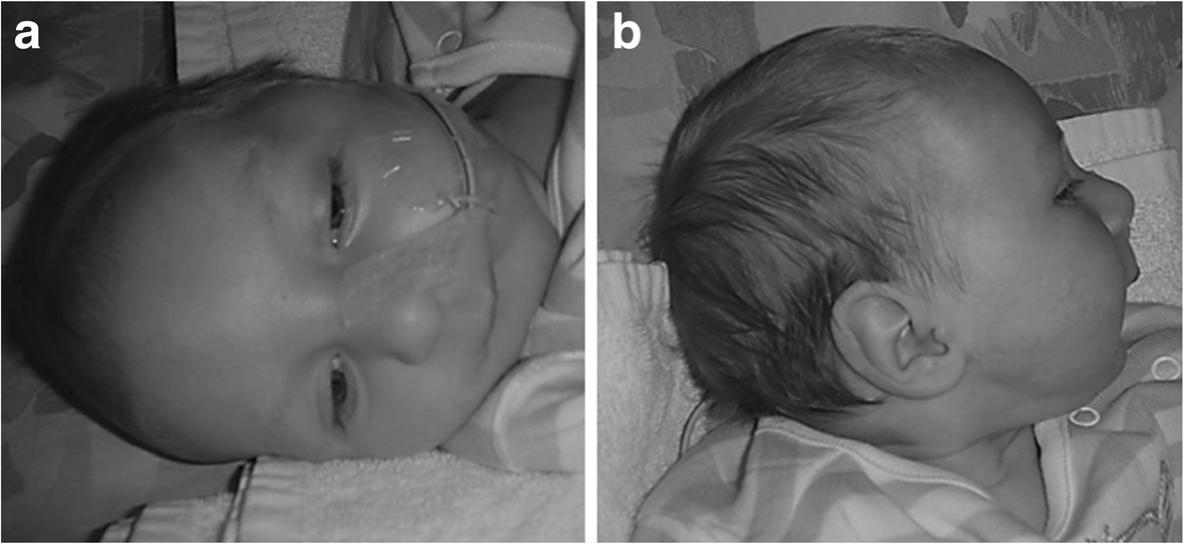
**Clinical features of patient 4 at the age of 3 months.** Note the overriding sutures, deeply positioned eyes and retrognathia, probably due to reduced fetal movements. The boy presents slight facial dysmorphism with hypertelorism, minor ptosis, posteriorly rotated ears, a prominent nose and thin upper lip.

Patient 4 is a 3 year old boy (Figure 3). He was born as the third child of non-consanguineous Belgian parents*.* Pregnancy was unsupervised. Vaginal delivery occurred, despite of breech presentation. He presented with severe hypotonia and generalized convulsions. Brain MRI at the age of 5 days was normal. Flexion contractures of knees and elbows were suggestive for reduced fetal movements. Clinical examination revealed a dry, scaly skin, campodactyly of the third and fourth finger, clinodactyly of the second and fifth finger and a micropenis with cryptorchidism. There was slight facial dysmorphism with low set, posteriorly rotated ears, a prominent nose with a broad root and retrognathia. The neck was short with loose and wrinkled skin. Feeding problems due to hypotonia and important gastroesophageal reflux made tube feeding necessary. Urosepsis at the age of 20 months led to the diagnosis of neurogenic bladder. Intermittent urinary catheterization and antibiotic prophylaxis prevented occurrence of further infections. Hearing tests and ophthalmological examination showed sensorineural deafness and cortical blindness respectively. The boy now presents with severe hypotonia, failure to thrive, progressive microcephaly, epilepsy and profound psychomotor retardation. There is still no head control. The general condition of the patient gradually declines.

### Glycoprotein analysis

**Figure 4:**
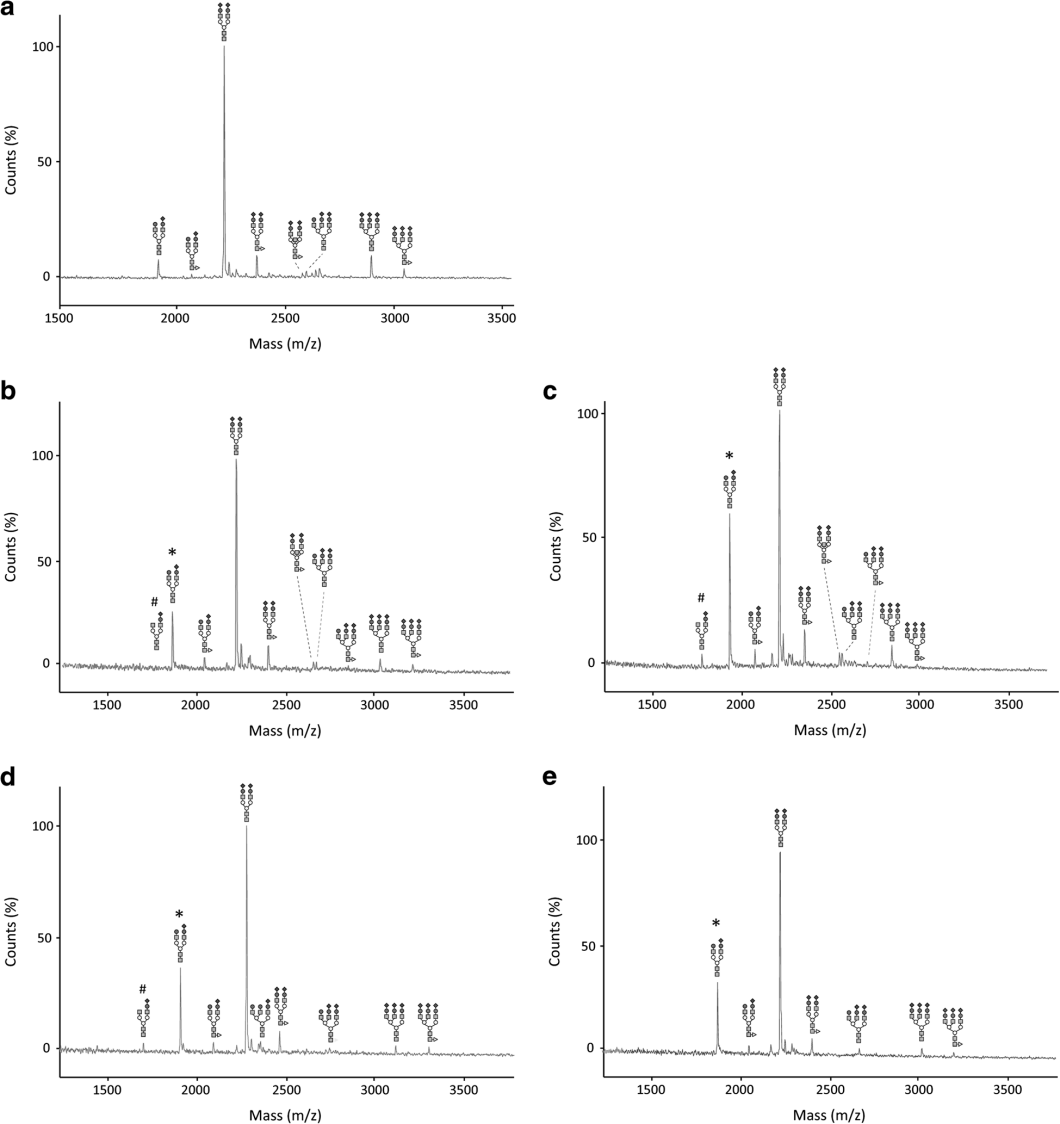
**Glycan analysis by MALDI-TOF MS.** Serum transferrin glycan analysis by MALDI-TOF MS in a control (**a**) and patients 1.1 (**b**), 2 (**c**), 3 (**d**), 4 (**e**). All the patients showed a defect in sialylation (*). Patients 1.1 (**b**), 2 (**c**) and 3 (**d**) also showed a slight deficient galactosylation (#). Symbols: square: N-acetylglucosamine; white circle: mannose; gray circle: galactose; diamond: sialic acid and triangle: fucose.

All patients showed a type 2 pattern on IEF of serum transferrin. MALDI–TOF MS of the N-glycans of serum transferrin suggested a defect in sialylation and in some of the patients a milder defect in galactosylation (Figure 4). These findings were compatible with a deficiency in the late Golgi glycosylation steps.

### Molecular analysis

Whole exome sequencing revealed a homozygous nonsense mutation in *COG5* (c.2518G > T; p.E840X) in patients 1.1 and 1.2. For patient 1.3 mutation analysis was not performed since no DNA sample was available. Sanger sequencing of *COG5* revealed two different mutations in patient 2 (c.556_560delAGTAAinsCT; p.S186_K187delinsL and c.95T > G; p.M32R). Patient 3 was found to be compound heterozygous for two other mutations (c.189delG; p.C64Vfs*6 and c.2338_2340dupATT; p.I780dup). Patient 4 was homozygous for a missense mutation at the 5^′^ boundary of exon 16 (c.1780G > T; p.V594F), causing a splice of exon 16.

The missense mutation p.M32R affects an amino acid that is embedded in a conserved region of the protein. To predict damaging effects of this missense mutation, the software tool Polyphen2 was used [22]. The missense mutation was predicted to be probably damaging with a score of 0.960. The c.556_560delAGTAAinsCT predicts an in frame deletion. The corresponding amino acid, as well as the flanking amino acids, are phylogenetically conserved. The c.2338_2340dupATT variant predicts an in frame insertion. The variant is not present in the dbSNP Database, or in the 1000 Genomes Project [23,24].

### Western blot analysis of the COG5 and COG7 subunits

**Figure 5:**
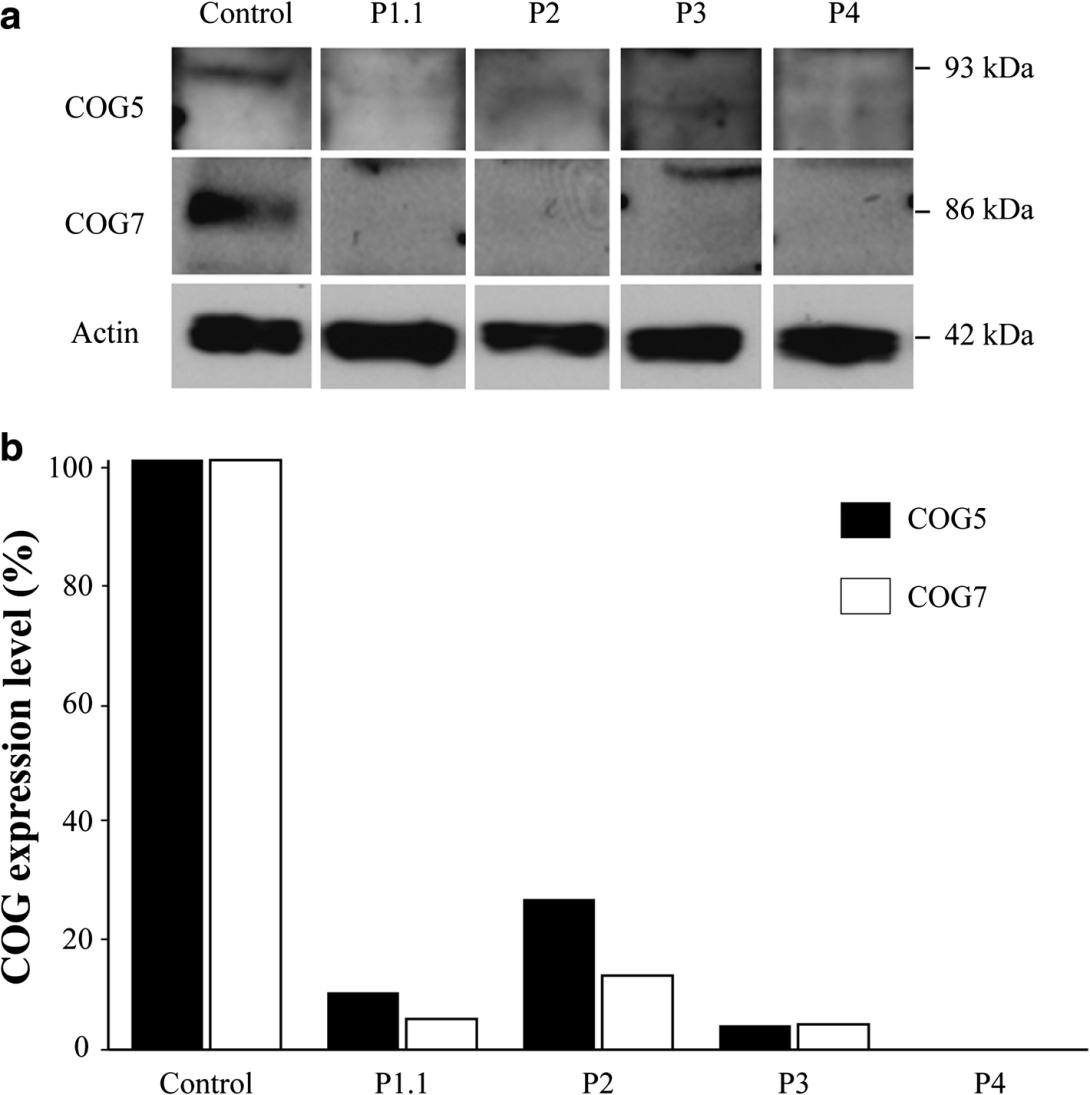
**Western blot analyses of COG5 and COG7.** Western blot analysis of the COG5 and the COG7 protein (**a**) and quantification of the steady state levels (**b**). Note the significant decrease in steady state levels of the COG5 and the COG7 protein in the patients compared to control.

To examine the impact of the mutations on the stability of COG5, western blot analysis in control and patients’ fibroblasts was performed. A significant decrease in steady state levels of the COG5 protein was found in our patients (0 to 25%), as compared to a healthy control. As stable subcomplex formation between COG5 and COG7 within lobe B is suggested, stability of the COG7 subunit was also checked. As expected, steady state levels of the COG7 protein were significantly reduced (0 to 13%), corresponding to results of previous studies in COG5-deficient mammalian cells (Figure 5). The steady state levels of COG5 and COG7 correlated with clinical severity.

### BFA treatment of cells

**Figure 6:**
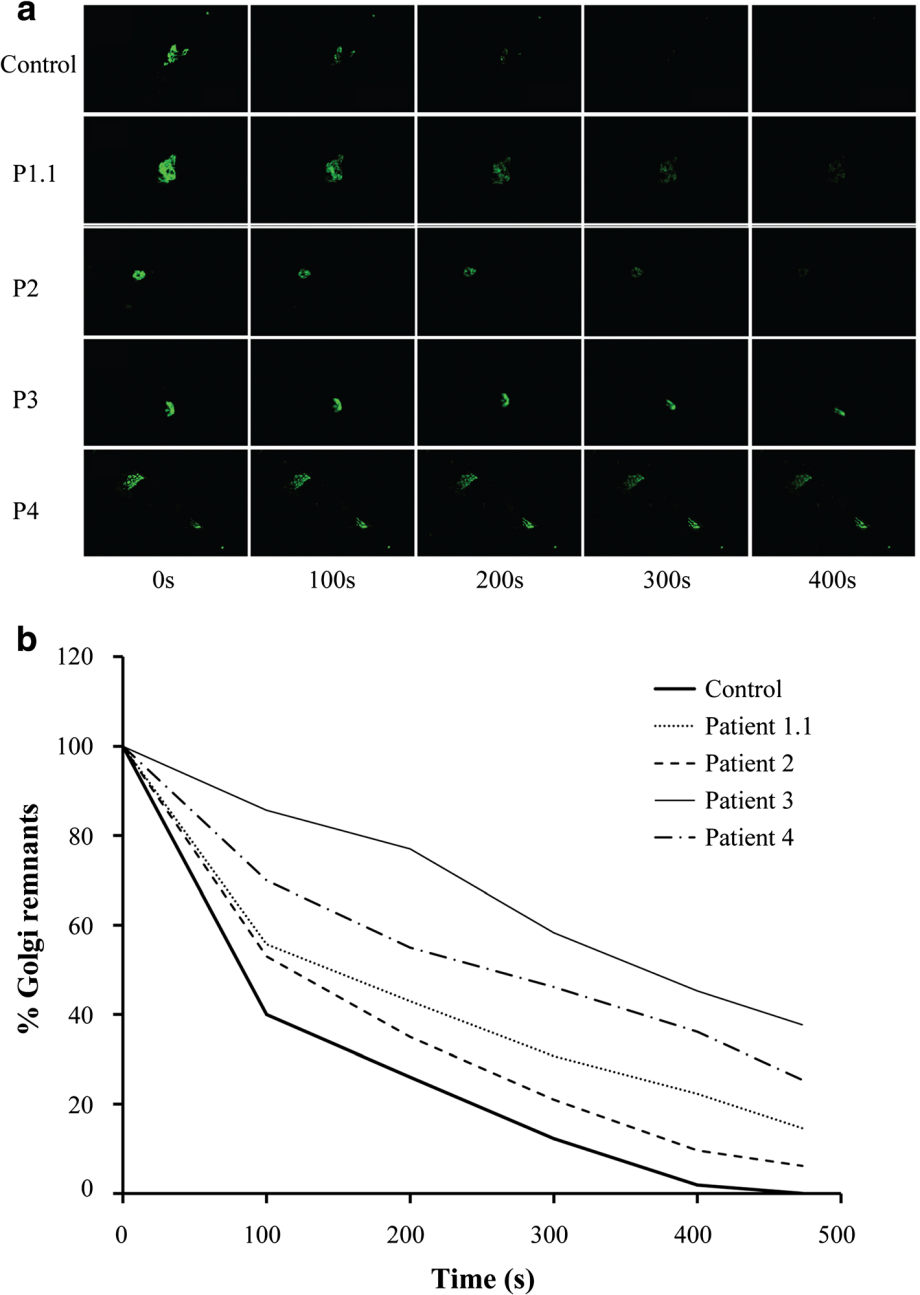
**BFA treatment of fibroblasts.** Effects of BFA on control and COG5 deficient cells, visualized by time-lapsed video microscopy (**a**). Quantification of the remaining fluorescence (**b**). Note the delay in the redistribution of GalT-GFP from the Golgi into the ER in COG5-deficient cells compared to control.

To study defects in retrograde trafficking, fibroblasts were first transfected with GalT-GFP and treated with BFA. Time lapse videomicroscopy was used to monitor the disappearance of GalT-GFP from the Golgi after the addition of BFA. A clear delay in the redistribution of the GalT-GFP into the ER was observed in COG5 deficient cells compared to control, as shown in Figure 6, after quantification of the remaining fluorescence. It has to be noted that this delay is different according to the observed mutations and suggests a correlation with clinical severity.

## Discussion

Most known CDG types are due to defects in genes involved in either the synthesis of the glycoconjugates, or of the sugar-donors. COG deficiencies cause CDG rather indirectly by affecting the trafficking and stability of the glycosylation machinery. Most of the mutations found in patients are localized in lobe B of the COG complex. Moreover, some of these mutations lead to complete loss of the lobe, while complete loss of lobe A has never been observed in CDG patients. This is consistent with studies performed in *Saccharomyces cerevisiae* and *Drosophila melanogaster* in which loss of lobe A is incompatible with life. However, from a clinical point of view most patients with COG-CDG, irrespective of the subunit affected, present a severe phenotype. Only a few mild/moderate cases are described [25].

In 2009 Paesold-Burda *et al.* published the first patient with COG5 deficiency. The girl presented only moderate mental retardation and a moderate delay in language and motor development with a progressive improvement in developmental milestones during childhood. We identified five additional patients with a COG5 deficiency. Mutation analysis was supported by a significant decrease in steady state levels of the COG5 protein on western blot and a delay in retrograde trafficking upon treatment of patients’ cells with BFA. Because recent studies in mammalian cells indicated that stable subcomplexes are formed between COG5 and COG7, steady state levels of the COG7 protein were investigated. A significant decrease of the COG7 protein level was detected [26].

Instead of a mild phenotype, we found a spectrum reaching from mild to very severe. Within this broad spectrum some common characteristics were present, i.e. hypotonia, microcephaly, some degree of mental retardation, a marked delay in language development or absence of speech and a short stature. Most patients showed slight facial dysmorphism with low set, posteriorly rotated ears, a relatively short neck with a low posterior hairline and a prominent nose. Interestingly, patients presenting with only mild mental retardation (e.g. patient 2) made progress with respect to their language and motor development, while patients on the opposite side of the spectrum, presenting with severe mental retardation, displayed further clinical deterioration (e.g. patient 4) or exhibited regression of previously acquired skills (e.g. patient 1.2 and 1.3). Furthermore, clinical features of patients on the severe end of the spectrum overlap with those of COG7-CDG, except for neonatal death. For example, patient 4 not only displayed loose, wrinkled and dry skin, but also developed seizures and neurogenic bladder dysfunction. Brain MRI 5 days after birth was normal. However, considering the progressive microcephaly global cerebral atrophy may be suspected. The clinical presentation of patient 3 also shows significant overlap with that of COG7-CDG. The patient presented with progressive microcephaly and significant global brain atrophy on MRI. He also suffered from significant liver involvement with cholestasis and neurogenic bladder dysfunction.

In conclusion, patients with COG5-CDG present different degrees of clinical severity. Since some of our patients show a clinical overlap with COG7-CDG, we hypothesize that interactions at protein level may be reflected in the phenotype.

## Abbreviations

BFA: Brefeldin A; CCD: COG Complex Dependent; CDG: Congenital Disorder of Glycosylation; COG: Conserved Oligomeric Golgi; ER: Endoplasmic Reticulum; IEF: Iso-Electric Focusing; IUGR: Intra-Uterine Growth Retardation; MALDI-TOF MS: Matrix Assisted Laser Desorption/Ionisation Time Of Flight Mass Spectrometry; MRI: Magnetic Resonance Imaging.

## Competing interests

The authors declare that they have no competing interests.

## Authors’ contributions

DR and JJ collected and compared the clinical data, and drafted the manuscript. LR, ND, CDV, CWF and JJ were involved in the clinical evaluation and follow-up of the patients. LS carried out the glycan analysis and the interpretation of the results. DR, LK, VR and GM carried out the molecular genetic studies and the interpretation of the results. CR and FF carried out the BFA assay, the Western Blot analysis and the interpretation of the results. All authors read and approved the final manuscript.
