# Correction: COG5-CDG: expanding the clinical spectrum

**DOI:** 10.1186/1750-1172-8-120

**Published:** 2013-08-14

**Authors:** Daisy Rymen, Liesbeth Keldermans, Valérie Race, Luc Régal, Nicolas Deconinck, Carlo Dionisi-Vici, Cheuk-wing Fung, Luisa Sturiale, Claire Rosnoblet, François Foulquier, Gert Matthijs, Jaak Jaeken

**Affiliations:** 1 Centre for Human Genetics, University of Leuven, Leuven, Belgium; 2 Centre for Metabolic Diseases, University Hospital Gasthuisberg, Herestraat 49, BE-3000, Leuven, Belgium; 3 University Children’s Hospital Queen Fabiola, Brussels, Belgium; 4 Division of Metabolism, Bambino Gesù Hospital, Rome, Italy; 5 Duchess of Kent Children’s Hospital, University of Hong Kong, Pokfulam, Hong Kong; 6 Institute of Chemistry and Technology of Polymers, Catania, Sicily, Italy; 7 Structural and Functional Glycobiology Unit, University of Lille 1, Lille 1, France

## Correction

After the publication of this work [1] it was brought to the authors attention that the appropriate ethical approval and patient consent was not stated in the manuscript. We regret any inconvenience that this may have caused. The correct statement is given below:

Following approval from the institutional ethics committee, the family of all patients in this study provided written consent to participate in this study, along with consent to publish their clinical details and photographs.
